# Global Biogeographic Distribution Patterns of Thermoacidophilic Verrucomicrobia Methanotrophs Suggest Allopatric Evolution

**DOI:** 10.3389/fmicb.2019.01129

**Published:** 2019-05-29

**Authors:** Helge-André Erikstad, Ruben Michael Ceballos, Natalie Bennett Smestad, Nils-Kåre Birkeland

**Affiliations:** ^1^Department of Biological Sciences, University of Bergen, Bergen, Norway; ^2^Department of Biological Sciences, University of Arkansas, Fayetteville, AR, United States

**Keywords:** *Methylacidiphilum*, biogeography, Verrucomicrobia, methanotrophs, acidophiles, PVC superphylum, allopatric evolution

## Abstract

Thermoacidophilic methane-oxidizing Verrucomicrobia of the candidate genus *Methylacidiphilum* represent a bacterial taxon adapted to highly acidic (pH 1–4) and moderate temperature (∼65°C) methane-containing geothermal environments. Their apparent ubiquity in acidic terrestrial volcanic areas makes them ideal model organisms to study prokaryotic biogeography. Three *Methylacidiphilum* species isolated from distantly-separated geothermal regions in Russia, New Zealand, and Italy were previously described. We have explored the intra-taxon phylogenetic patterns of these organisms based on comparative genome analyses and phenotypic comparisons with six new Verrucomicrobia methanotroph isolates from other globally-separated acidic geothermal locations. Comparison of rRNA and particulate methane monooxygenase (*pmoCAB*) operon sequences indicates a close phylogenetic relationship among the new isolates as well as with the previously characterized strains. All share similar cell morphology including the presence of extensive intracellular inclusion bodies and lack of intracellular membrane systems, which are typical for proteobacterial methanotrophs. However, genome sequence comparisons and concatenated MLST-based phylogenetic analyses separate the new isolates into three distinct species-level groups. Three recently processed isolates from the Azores (each from geographically-separate hot springs *within* the region) and a single isolate from Iceland are highly similar, sharing more than 88% *in silico* genome homology with each other as well as with the previous isolate, *Methylacidiphilum fumariolicum* strain SolV, from Italy. These appear to constitute a distinct European/Atlantic clade. However, two of the new isolates – one from the Yellowstone National Park (United States) and another from The Philippines – constitute separate and novel *Methylacidiphilum* species. There is no clear correlation between fatty acid profiles and geographic distance between origins, or any phylogenetic relationship. Serological analysis using antiserum raised against *M. kamchatkense* strain Kam1 revealed large differences in the degree of cross-reactivity with no correlation with other factors. However, the genetic distance between the strains does correlate to the distance between their geographic origins and suggests a global biogeographic pattern shaped by an isolation-by-distance mechanism. These results further confirm terrestrial geothermal springs as isolated islands featuring allopatric prokaryotic speciation.

## Introduction

Microbial biogeography and mechanisms for speciation among microbes are still controversial and poorly understood topics. In general, prokaryotes are considered to be cosmopolitan. Specifically, it is thought that they exhibit global distribution without clear biogeographic boundaries. The long-standing Baas-Becking postulate, “Everything is everywhere, but the environment selects” (Baas-Becking, 1934), has been a cornerstone hypothesis for the evolution and diversification of prokaryotes for decades. A number of studies provide support for the cosmopolitan nature of prokaryotes. Identical microorganisms have been recovered from environments across the globe. Examples from marine (Massana et al., 2000), freshwater (Glöckner et al., 2000), and soil (Brewer et al., 2017) systems have been shown to be globally distributed and lack of any notable biogeographic structure. Furthermore, studies of bacterial pathogens and endospore formers also appear to demonstrate cosmopolitan distribution (Musser et al., 1990; Smith et al., 1991; Roberts and Cohan, 1995). Even prokaryotes recovered from deep subsurface environments, in some cases, have been shown to be virtually identical with isolates derived from widely-separated geographic regions. For example, an isolate of a thermophilic sulfate reducer belonging to the species *Thermodesulfobacterium thermophilum* sampled from hot water produced from deep oil wells in the North Sea is serologically indistinguishable from those found near the Caspian Sea (Christensen et al., 1992). A strain of the archaeal sulfate reducer, *Archaeoglobus fulgidus*, from a shallow hot vent in the Mediterranean Sea (Stetter, 1988), is almost indistinguishable from an isolate derived from an oil well in the North Sea (Beeder et al., 1994; Birkeland et al., 2017). Likewise, *Thermosipho africanus* strain TCF52B, isolated from a North Sea oil well (Dahle et al., 2008), is virtually identical with *T. africanus* strain Ob7^T^, isolated from a shallow hot vent in Djibouti, Africa as determined by genomic DNA-DNA hybridization (DDH) and 16S rRNA gene sequence analyses (Huber et al., 1989; Dahle et al., 2008). All three of these examples support the concept of a cosmopolitan distribution of prokaryotes and the idea that geographic distance plays a minor role in speciation and evolution in prokaryotic systems.

However, during the last 15 years, conspicuous patterns of biogeographic structure in global distribution of microbes have been reported. This has been shown for members of the thermophilic archaeal genus, *Sulfolobus*, both by comparison of selected chromosomal loci (Whitaker et al., 2003) and complete pan-genomes (Reno et al., 2009). These organisms are adapted to acidic terrestrial hot springs, and do not possess any spores or spore-like structures that can facilitate dispersion across large geographical distances. Isolates from similar hot springs as far apart as Kamchatka (Eastern Russia), Iceland, and the United States formed distinct phylogenetic clades, indicating evolution through local adaptation or random genetic drift with no evidence of gene flow between the isolated populations. Interestingly, the question of whether biogeographic structure exists in a common virus of the genus – namely, the *Sulfolobus* Spindle-shaped Virus – and the functional implications (e.g., infectivity) has been controversial (Snyder et al., 2007; Held and Whitaker, 2009; Ceballos et al., 2012). Terrestrial geothermal features represent environments with highly specific physicochemical conditions, often with extreme temperature and pH values. Dispersion of microorganisms between distantly located hot springs therefore seems highly unlikely, and such environments are thus regarded as isolated islands with strong geographic barriers. Select extremophiles from hypersaline lakes and mines (e.g., *Nocardiopsis* spp.) have also revealed a strong influence of spatial distance on the shaping of location-specific phylotypes of bacterial populations, especially across large spatial scales (He et al., 2015).

Three thermoacidophilic methanotrophs belonging to the phylum Verrucomicrobia, which have been given the provisional genus name *Methylacidiphilum*, have been isolated from acidic terrestrial geothermal environments in New Zealand, Italy, and Kamchatka, Russia (Dunfield et al., 2007; Pol et al., 2007; Islam et al., 2008). These organisms are extremely acidophilic (and moderately thermophilic) with optimal pH and temperatures for growth of 2.0–3.5 and 55–60°C, respectively (Op den Camp et al., 2009). Together with a mesophilic sister group, which is given the provisional genus name *Methylacidimicrobium*, these thermophilic methanotrophic Verrucomicrobia constitute a novel subdivision within the phylum Verrucomicrobia (Van Teeseling et al., 2014). In this study, we introduce six additional thermoacidophilic methanotrophic Verrucomicrobia isolates from acidic hot springs in: Iceland, Yellowstone National Park (United States), the Azores, and the Philippines. Together with previously described strains, a collection of nine isolates from closely located sampling sites from a few kilometers to 3000 km apart as well as from more distantly located sites (i.e., > 18,000 km apart) are available. Using genomic comparisons, our data demonstrate a clear correlation between the distance of the origin and relationship between strains, supporting a distance dependent speciation.

## Materials and Methods

### Sampling, Isolation, and Cultivation

Sampling was done by collecting a slurry of water and top layer of sediment (to < 3 cm depth) from shallow (5–20 cm deep) sites of acidic hot springs in The Azores (Furnas, Ribeira Grande and Furnas das Lagos), Iceland (Krysuvik), The Philippines (Makiling mud spring) and Yellowstone National Park (the Norris geyser basin), United States. Characteristics and geographic coordinates of the springs are provided in Table 1, including time of sampling and strain designations. The chemical composition of spring waters is given in Supplementary Table S1. The distance between sampling sites were calculated using Google Maps^1^. This is based on shortest distance between points defined by latitude:longitude coordinates. Inoculation and enrichment was done using a 10-fold dilution of a standard methanotrophic ammonium-based mineral medium (Whittenbury et al., 1970) adjusted to pH 3.5 and prepared as previously described (Islam et al., 2008; Erikstad et al., 2012). Enrichment cultures were prepared by inoculation of 1 ml sample slurry to 10 ml medium in 120 ml serum flasks which subsequently were closed with a butyl rubber cap and an aluminum crimp seal. Methane was added by a syringe to 20% headspace concentration. Flasks were incubated at 55°C with shaking at 150 rpm. Growth was monitored by phase contrast microscopy, using a Nikon, Eclipse E400 microscope. CH_4_ consumption was analyzed in a Hewlet Packard 6890 gas chromatograph equipped with a 1.83 m Haysep R column (80/100 mesh) and a thermal conductivity detector (150°C). After verification of growth and methane consumption, isolation was done by two times dilution to extinction. All isolates were identified as methanotrophic Verrucomicrobia by PCR and sequencing of their 16S rRNA genes as earlier described (Islam et al., 2008). All subsequent cultivations were performed in the medium and under conditions as described above. Since 2015 the medium has been supplemented with 300 nM CeCl_3_ as it was shown that lanthanides strongly enhance growth of these microorganisms (Pol et al., 2014).

**Table 1:** Overview of the characteristics and location of the sampling sites from which the novel methanotrophic isolates were recovered.

Origin	Time of sampling	Strain name	Temperature	pH	Coordinates
Furnas, The Azores	September 2010	Fur	55°C	2	N37°46.385′W025°18.21′
Ribeira Grande, The Azores	May/June 2012	Rib	62°C	0–1	N37°48.155′W025°29.16′
Furnas das Lagos, The Azores	May/June 2012	Fdl	62–64°C	2–3	N37°46.127′W025°19.89′
Yellowstone National Park, United States)	September 2011	Yel	50°C	2.8	N44°43.890′W110°42.682′
Krysuvik, Iceland	May, 2013	Ice	60°C	3	N63°53.278′W022°03.405′
Makiling mud spring, The Philippines	2014	Phi	55–65°C	3	N14°8.191′E121°13.262′

### Electron Microscopy

Transmission electron microscopy was performed as described previously (Islam et al., 2008). Briefly, harvested cells were fixed in 2.5% glutaraldehyde at 4°C overnight followed by 3 washes in 0.25% NaCl for 15 min each before post-fixation with 1% OsO_4_ for 60 min. Then, cells were washed and dehydrated in increasing concentrations of ethanol before being embedded in Agar 100 resin. Thin sections were prepared using a Reichert Ultracut ultramicrotome and stained with lead and uranyl before being examined with a Jeol JEM-1230 electron microscope.

For scanning electron microscopy, 2 ml samples of six to 7 days old cultures were removed from serum bottles with a 10 ml BD syringe with a Luer-Lok^TM^ tip. Polycarbonate membrane filters (Millipore Isopore^TM^, 0.2 μm pore size) were inserted into 13 mm Millipore Swinnex filter holders by the use of forceps, and then mounted onto the syringes containing the cultures. The cultures were filtrated through the filters, which were subsequently washed with cacodylate buffer (pH 7.4) before being fixed in 6% glutaraldehyde in cacodylate buffer for 2 h. The filters were then washed with 0.5 ml cacodylate buffer 10 × 5 min and distilled H_2_O for 5 × 5 min. Dehydration of the cells were then performed by step-wise treatment with 50, 75, 96% and finally 2 × 100% ethanol. Filters were incubated for 15 min in each step and then transferred to a Quorum Technologies Critical point dryer CPD7501. The filters were first flushed with 100% liquid CO_2_ three times using a pressure between 700 and 900 psi at -20°C to 4°C. A critical point was then reached by increasing the pressure to 1300–1500 psi and the temperature to 35–37°C. The filters were then mounted onto aluminum stubs with double sided carbon tape and coated with a 10 nm thick layer of gold palladium in a sputter coater (Jeol JFC-2300HR High resolution fine coater). The samples were then examined in a Jeol JSM-7400F scanning electron microscope.

### Genome Sequence Analysis and Phylogeny

Genomic DNA was extracted with the use of a GenElute^TM^ Bacterial Genomic DNA kit following the manufacturer’s instructions and sequenced at GATC Biotech, Germany^2^ using Illumina paired-end technology. A total of 11,700 to more than 28,000 reads accounting for 1.5–3.6 Giga bases were obtained. Assembly by CLC Genomics Workbench 8.5.1 resulted in 2.3–2.4 Mega bases of unique sequence data distributed into 58–113 contigs. Annotation was performed using RAST^3^ and the NCBI Prokaryotic Annotation Pipeline^4^. The whole-genome shotgun projects have been deposited at DDBJ/EMBL/GenBank under the accession numbers LXJS00000000 (Strain Fur), LXNL00000000 (Strain Fdl), LXNK00000000, LXOX00000000 (Strain Ice), LXQC00000000 (Strain Phi) and LXQB00000000 (Strain Yel). Pairwise genome sequence similarities were calculated using the *in-silico* Genome-To-Genome Distance Calculator available at DSMZ^5^. Contigs obtained from the *de novo* assembly were ordered and aligned using the *M. fumariolicum* SolV genome (Anvar et al., 2014) as template by the use of MAUVE version 2.4.0^6^ (Darling et al., 2004).

The evolutionary history of *Methylacidiphilum* strains was inferred using sequence concatemers of the genes *gyrA, gyrB, ftsZ, rho, dnaG, rpoE, and groEL*, by using the Maximum Likelihood method based on the Tamura-Nei model (Tamura and Nei, 1993). Reference genes were extracted from the complete genome sequences of *M. fumariolicum* SolV (Anvar et al., 2014), *Methylacidiphilum infernorum* strain V4 (Hou et al., 2008) and the draft sequence of *Methylacidiphilum kamchatkense* strain Kam1 (Erikstad and Birkeland, 2015). The mesophilic methanotrophic verrucomicrobial isolates, *Methylacidimicrobium fagopyrum* 3C (Van Teeseling et al., 2014; BioProject PRJNA165235) and *Methylacidimicrobium* sp. LP2A (Op den Camp et al., 2009; Acc. no. JAFS01000000), were also included. Initial tree(s) for the heuristic search were obtained automatically by applying Neighbor-Join and BioNJ algorithms to a matrix of pairwise distances estimated using the Maximum Composite Likelihood approach, and then selecting the topology with superior log likelihood value. All positions containing gaps and missing data were eliminated. The analysis was conducted in MEGA7 (Kumar et al., 2016).

From DNA sequence alignments of select genes (e.g., *pmoAB2*), identity matrices were constructed to illustrate pairwise comparisons of sequence similarities between different strains. In addition, a geographic distance matrix was constructed, which denotes physical separation (km) between sites from which each isolate was derived. Using a “genetic distance” matrix with a geographic distance matrix, a Mantel test was employed to compute correlation between the two distance matrices. The resulting test statistic, *r_M_*, is defined as:

$$\begin{array}{c} r_{\text{M}}=\frac{1}{d-1}\sum_{\text{i}=1}^{\text{n}-1}\sum_{\text{j}=\text{i}+1}^{\text{n}}stand(D_{\text{genetic}})ij\;stand(D_{geographic})ij \end{array}$$

where $d=\frac{\left(n(n-1\right)}{2}$ and *n* is the number of rows/columns (i.e., 9) in each of the distance matrices (Mantel, 1967). Possible values for *r_M_* from -1 to +1 indicate the presence of either a negative or positive correlation (or absence of correlation if *r_M_* = 0) for genetic distance versus geographic distance. To test for potential correlation between genetic divergence and tectonic plate sympatry, the Spearman correlation method was employed (Dodge, 2008). The Spearman rank correlation (r_s_) is defined by:

$$r_{s}=\frac{cov(rg_{\text{genetic}},\;rg_{\text{tectonic}})}{\sigma_{rg}{}_{{}_{genetic}}\sigma_{rg}{}_{{}_{tectonic}}},$$

where *cov*(*rg*_genetic_, *rg*_tectonic_) is the covariance of the rank variables and σ_rg_genetic__ and σ_rg_tectonic__ are the standard deviations of the rank variables.

### Fatty Acid Analysis

Analysis of the cellular fatty acid composition was determined by Leibniz Institute DSMZ-German collection of Microorganisms and Cell Cultures^7^. Fatty acid methyl esters obtained from freeze-dried cell pellets by saponification, methylation and extraction using minor modifications of a method previously described (Miller, 1982; Kuykendall et al., 1988). The fatty acid methyl ester mixtures were separated using Sherlock Microbial Identification System (MIS) (MIDI, Microbial ID, Newark, DE 19711, United States) which consisted of an Agilent model 6890N gas chromatograph fitted with a 5% phenyl-methyl silicone capillary column (0.2 mm × 25 m), a flame ionization detector, Agilent model 7683A automatic sampler, and a HP-computer with MIDI database (Hewlett-Packard Co., Palo Alto, CA, United States). Peaks are automatically integrated and fatty acid names and percentages calculated by the MIS Standard Software (Microbial ID). The gas chromatographic parameters were as follows: carrier gas, ultra-high-purity hydrogen; column head pressure 60 kPa; injection volume 2 μl; column split ratio, 100:1; septum purge 5 ml/min; column temperature, 170–270°C at 5°C/min; injection port temperature, 240°C; and detector temperature, 300°C.

### Serology

Cells from an actively growing culture of Kam1 were harvested by centrifugation and washed twice in phosphate buffered saline (PBS), pH 7, containing 3.7% formaldehyde, and washed twice in PBS. The final resuspension contained 5 × 10^9^ cells per ml and was shipped on ice to Biogenes (Germany)^8^ for production of polyclonal antibodies in rabbits through intramuscular injections. The rabbits were first injected with 1 ml antigen followed by weekly injections with 0.5 ml antigen for 3 weeks. Two months later, a boost of 1 ml was injected followed by final bleeding 1 week later. Pre-immunserum was collected prior to starting the immunization programme.

ELISA was performed on freeze-dried pellets of our bacterial isolates. The 96- well immunoplate (Nunc MaxiSorp^TM^) was coated with 150 μl of the antigen solution overnight at 4°C (10 μg/ml in PBS- buffer). The antigens were not applied to the outermost wells. Skimmed milk powder (5 μg/ml) was used as a negative control. All the following washing steps was done with x3 PBS-Tween buffer (PBS-T), pH 7.4 (200 μL to each well). After coating, the wells were washed and subsequently blocked with a 3% skimmed milk powder blocking solution in PBS-T for 1 h at room temperature (200 μL to each well) and followed by additional washes. The primary antiserum was initially diluted 1:400 in PBS-T followed by consecutive to-fold dilutions per well (200 μL to each well in total). The negative control serum was initially diluted 1:100 in PBS-T otherwise the same dilution pattern as for the primary antiserum. After incubating with primary antiserum at room temperature for 2 h, the plate was washed and added 50 μl pr well of 1:3000 diluted “goat anti-rabbit” Ig conjugate (diluted in PBS-T), followed by another incubation at room temperature for 1 h. Fifty microliter of peroxidase substrate solution, o-Phenyleneidamine (OPD), was added to each well. After 6 min, 50 μl of 2.5N H_2_SO4 was added each well to stop the substrate reaction. Titertek Multiscan spectrophotometric microplate reader was used to read the results at OD_492_ nm.

## Results

### Isolation and Initial Characterization

**FIGURE 1:**
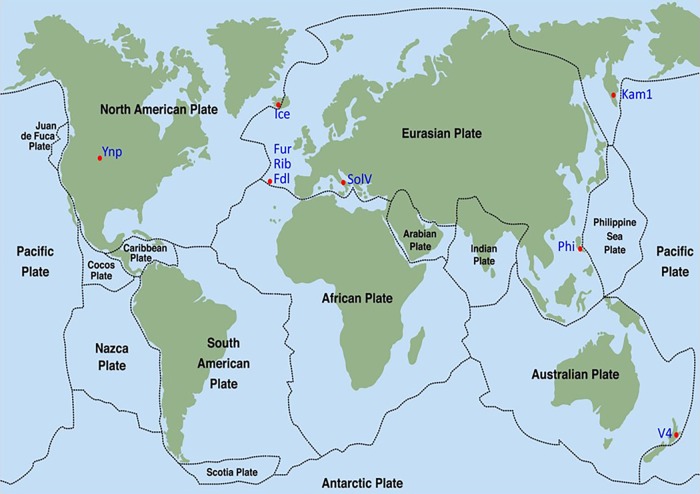
Sampling sites for isolation of thermoacidophilic methanotrophic Verrucomicrobia (indicated by red dots). Strain designations are indicated by blue color. Strippled lines indicate boundaries between tectonic plates.

The six methanotrophic isolates presented here, belonging to the phylum Verrucomicrobia, were isolated from geothermal environments across the globe (Figure 1 and Table 1). In addition, *M. kamchatkense* strain Kam1, which was previously isolated by our lab (Islam et al., 2008), was included in this study. Three of the new isolates originate from three different locations on the San Miguel Island in The Azores. The isolates are designated according to their respective sampling sites: Furnas (Fur), Ribeira Grande (Rib), and Furnas Das Lagos (Fdl). Temperatures at sample sites located in the Azores ranged from 55 to 64°C and pH from 0 to 3. The isolate from Yellowstone National Park (Yel) was collected in the Norris geyser basin from a site at 50°C and pH 2.8. We collected the Iceland isolate (Ice), from a spring at 60°C and pH 3, which was located in the Seltun area in the Krysuvik geothermal field. The isolate from the Philippines was recovered from the Mount Makiling mud spring in the Laguna province on the island of Luzon. This sampling site was between 55 and 65°C and pH 3.

**FIGURE 2:**
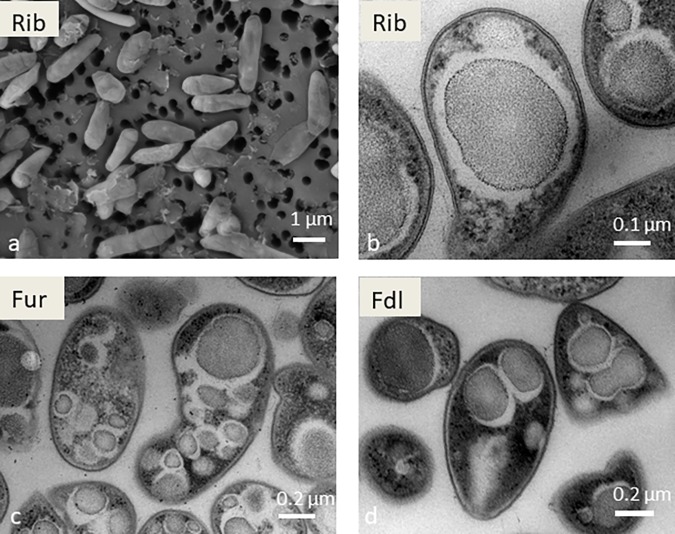
Scanning electron microscopy image of strain Rib **(a)** and transmission electron microscopy images of strains Rib **(b)**, Fur **(c),** and Fdl **(d)**. Scale bars are shown at the bottom right in all images.

Morphologically and physiologically, the six strains are very similar. Optimal growth was at 53–57°C and pH 2.5–3.5 (data not shown). Scanning electron microscopy images of the isolates confirmed that the cells are short rods, on average about 1–2 μm in length and 0.5 μm in diameter (Figure 2). Under transmission electron microscopy (TEM), multiple electron-dense inclusions, which are suspected stores of glycogen, are visible in all of the new isolates (Figure 2). These morphological and physiological data are consistent with previous reports. For example, in strain SolV similar inclusions were observed and were subsequently shown to contain glycogen (Khadem et al., 2012).

### Genome Analysis and Comparison

**FIGURE 3:**
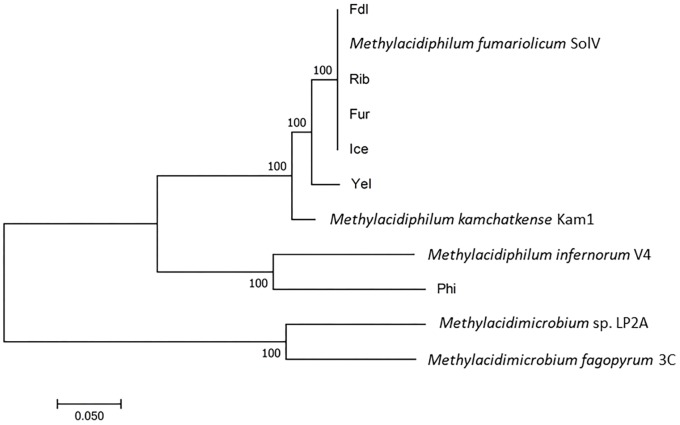
Phylogenetic analysis of *Methylacidiphilum* and *Methylacidimicrobium* strains by the Maximum Likelihood method using concatemers of seven housekeeping genes. The percentage of trees in which the associated taxa clustered together is shown next to the branches. The tree is drawn to scale, with branch lengths measured as the number of substitutions per site. Bootstrap values are indicated at nodes. All positions containing gaps and missing data were eliminated.

Like the three previously characterized strains, all of the six new isolates contain a single ribosomal RNA operon. All of the nine isolates are phylogenetically closely related, sharing ≥98.6% 16S rRNA sequence identity, which is clearly above the generally accepted species threshold value of 97% (Stackebrandt and Goebel, 1994) but close to the recently suggested species cutoff value of 98.7% (Chun et al., 2018). A phylogenetic tree based on concatenated sequences of seven housekeeping genes (*gyrA, gyrB, ftsZ, rho, dnaG, rpoE*, and *groEL*) illustrates that the European isolates group into a European clade with strain Kam1 and strain Yel as the closest relatives. *Methylacidiphilum infernorum* strain V4 (from New Zealand) and strain Phi appear to be phylogenetically distinct species (Figure 3), as also supported by the 16S rRNA tree (Supplementary Figure S1). Given the biogeographic boundaries of isolates from New Zealand (V4) and the Philippines and their relative proximity to one another when compared to the distance between these mid-to-south Pacific islands to Europe, the phylogenetic relationships are convincing. Moreover, *in silico* genome homology analyses (i.e., DNA-DNA hybridization or DDH) reveal that the strains Yel and Kam1 may also be regarded as separate species since DDH values were below the 70% DNA-DNA threshold line (see Table 2) considered to represent distinct species (Wayne et al., 1987). This is also supported by the pairwise average nucleotide identity (ANI) values (Supplementary Table S2). The European isolates (strains: Ice, Fur, Rib, Fdl, and *Methylacidiphilum fumariolicum* strain SolV) share more than 88% *in silico* gene homology while strains Phi and V4 only share less than 21.6% gene homology to the other strains.

**Table 2:** Pairwise genomic DNA-DNA *in silico* hybridization values (in bold) and 16S rRNA sequence identities of all thermoacidophilic methanotrophic Verrucomicrobia isolates.

Strain designation	SolV	Fur	Rib	Fdl	Ice	Yel	Phi	V4	Kam1
SolV	**100**								
	100								
Fur	**89.1**	**100**							
	100	100							
Rib	**89.4**	**100**	**100**						
	100	100	100						
Fdl	**89.7**	**100**	**100**	**100**					
	100	100	100	100					
Ice	**88.4**	**98.9**	**99**	**100**	**100**				
	100	100	100	100	100				
Yel	**55**	**60.7**	**60.7**	**55.4**	**61**	**100**			
	99.5	99.5	99.5	99.5	99.5	100			
Phi	**17.5**	**17.6**	**17.6**	**21.6**	**21.6**	**20.1**	**100**		
	98.7	98.7	98.7	98.7	98.7	98.7	100		
V4	**17.1**	**17.1**	**17.1**	**17.1**	**19.6**	**20.4**	**20.1**	**100**	
	98.6	98.6	98.6	98.6	98.7	98.7	99.2	100	
Kam1	**49.8**	**54.8**	**54.8**	**49.9**	**49.9**	**50.4**	**20.8**	**17.1**	**100**
	99.7	99.7	99.7	99.7	99.7	99.5	98.7	98.7	100

**FIGURE 4:**
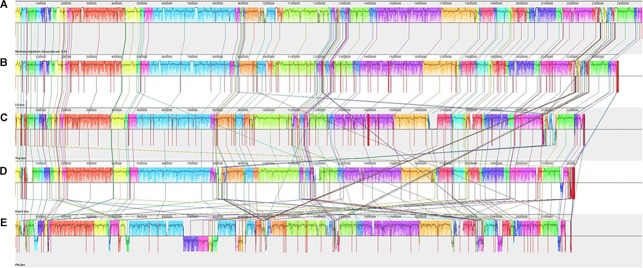
Alignment of the contigs of *Methylacidiphilum* spp. strains Fdl **(B)**, Yel **(C)**, Kam1 **(D)**, and Phi **(E)** against the *Methylacidiphilum fumariolicum* SolV **(A)** chromosome using MAUVE 2.4.0. Vertical red lines indicate borders between contigs.

After reordering of genome contigs of strains Fdl, Yel, Phi, and Kam1 against the completed *M. fumariolicum* SolV genome (Anvar et al., 2014), all the new strains aligned reasonably well with the SolV genome, indicating a large degree of gene synteny among the genomes (Figure 4) despite a very low overall genome sequence similarity between some strains. Larger reorganizations and inversions are only seen for strain Phi. A few larger regions of unique non-homologous sequences can also be discerned in strains Kam1, Yel, and Phi. As compared to SolV, the four new genomes appear to be somewhat shorter, but a firm conclusion should await a complete PacBio sequence analysis.

All of the six new isolates possess Rubisco-encoding genes, and thus appear to assimilate carbon from CO_2_ like has been shown for strain SolV (Khadem et al., 2011), as well as a complete set of nitrogen fixation genes. Strains Phi, Fur, and Fdl carry three copies of the *pmo*CAB genes like the three other described *Methylacidiphilum* species, while in strain Rib and Ice, only two complete *pmo*CAB operons could be identified from the draft genome sequences in addition to an apparently truncated *pmo*AB cluster. In strain Yel, only one complete *pmo*CAB cluster was identified, and two truncated *pmo*AB and *pmo*CA clusters. All strains contain one methanol dehydrogenase gene encoding the lanthanide-dependent XoxF type as described for SolV (Pol et al., 2014).

**FIGURE 5:**
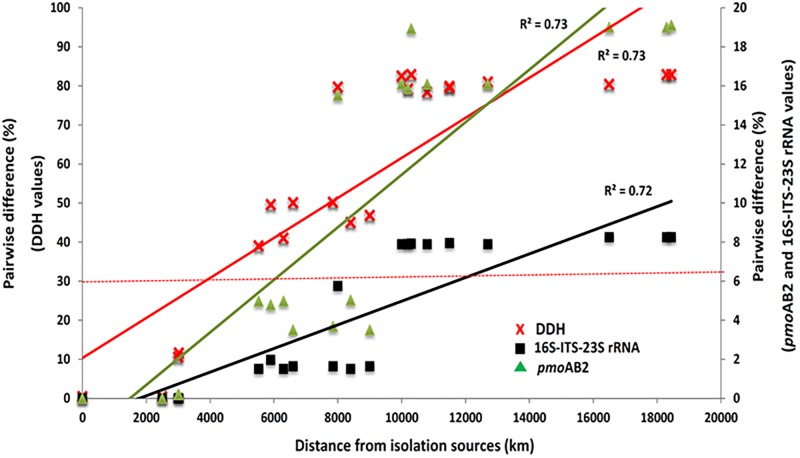
Scatter plot of pairwise nucleotide differences of the 16S-ITS-23S rRNA (black squares) and *pmo*AB2 (green triangles) gene sequences of the *Methylacidiphilum* spp. isolates and pairwise genome sequence differences (red crosses) based on *in silico* DNA-DNA hybridization (DDH) values. The left y-axis indicates genomic differences while the right y-axis indicates 16S-ITS-23S rRNA and *pmo*AB2 differences. The x-axis indicates the geographic distance between the isolation source for each pairwise strain comparison. A stippled demarcation line for DDH threshold for species separation (>30% difference or 70% > similarity) is included.

**FIGURE 6:**
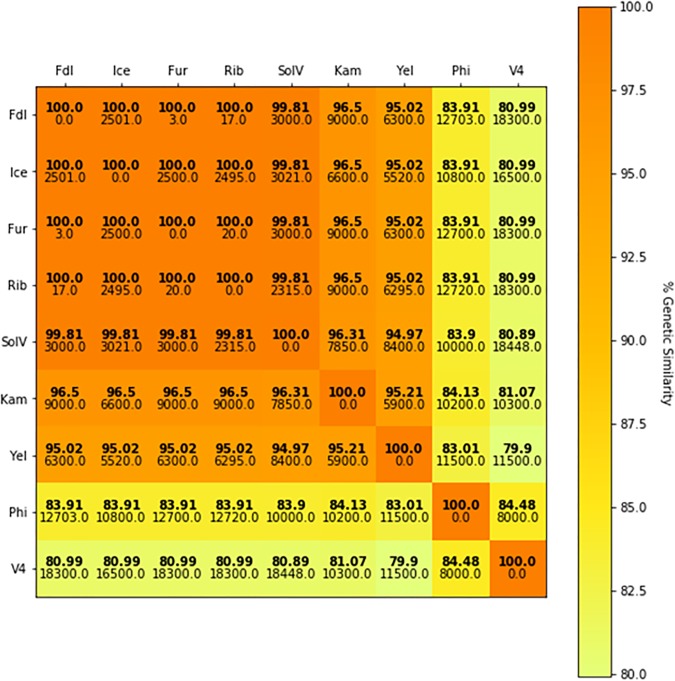
*pmo*AB2 sequence identity matrix. The color in the heat map represents percent sequence identity (%) for *pmoAB2* across isolates (bold) with darker colors indicating higher sequence similarity between two isolates and lighter colors representing less sequence similarity. Geographic distances (km) between habitats from each pair of comparisons is shown below the genetic identity value. The Mantel statistic (r_M_) shows a significant correlation between genetic divergence and geographic distance (r_M_ = 0.885, *p* < 0.001).

When internal transcribed spacer regions (ITS) and segments of the 23S rRNA sequence were included in the phylogenetic analysis, percent identity is somewhat reduced (≥91.75%) but remains above 90% (Figure 5). Comparisons made with a phylum-specific *pmoA* marker (i.e., *pmoA2*), sequence similarities are ≥79.9% (see Figure 5). Based on ongoing work in hyperthermophilic archaea and the hypothesis that there is correlation between genetic similarity and tectonic plate sympatry, the Mantel test was used to test for correlations between genetic divergence (among isolates) and geographic distance (i.e., distance between habitats from which isolates were derived). Alternatively, to test potential correlation between genetic divergence and tectonic sympatry, the Spearman correlation method (i.e., *r_s_* statistic) was employed. A positive correlation between genetic “distance” versus geographic distance emerged from the Mantel test (r_M_ = 0.885, *p* < 0.001) (Figure 6). In addition, the Spearman analysis was able to support a relationship between genetic similarity and tectonic plate sympatry (*r_s_* = 0.532, *p* < 0.001, *N* = 9).

### Chemotaxonomy and Serology

The PLFA profiles of the six new isolates and strain Kam1 displayed a dominance of saturated phospholipids (Supplementary Table S3). PLFAs i14:0, a15:0, and 18:0 account for more than 61% of the total PLFA content. Only trace amounts of unsaturated PLFAs: a13:1 12–13; 18:1 ω9c; and, 18:1 ω7c – were detected. Overall, the major PLFAs were present across most strains with some variability in abundances. One notable exception was the Icelandic strain (Ice), which exhibited a different PLFA profile: 15:0; i16:0; 16:0; 16:0 3OH; and, 17:0.

Antiserum raised against strain Kam1 showed strong strain-specificity in an ELISA experiment using whole cells of the isolates as antigens at high antiserum dilutions (≥51,200-fold) (Supplementary Figure S2). This demonstrates the presence of highly specific cell surface antigens in Kam1 as compared to the other isolates. The antigenic determinant is not known, but it might be LPS. At lower antiserum dilutions considerable cross-reactions can be observed, which are most likely caused by unspecific binding reactions.

## Discussion

Methane from natural sources comprises an estimated ∼36% of total global CH_4_ emissions, while anthropogenic activities account for ∼64% (Bousquet et al., 2006). Fossil fuel use and farming activity have resulted in the doubling of methane emissions over the past 150 years (IPCC, 2007). Microbiological mitigation of natural methane release is accomplished, in part, by several taxonomic groups of methanotrophs (see review by Op den Camp et al., 2009). Although emissions from geothermal features worldwide may only account for up to 1% of the total methane release (Etiope and Klusman, 2002), methanotrophy by mesophilic and/or neutrophilic *Alphaproteobacteria* and *Gammaproteobacteria* of three well-established families: *Methylocystaceae* (α-proteobacteria), *Beijerinckiaceae* (α-proteobacteria), and *Methylococcaceae* (γ-proteobacteria) – is limited at extremes of low pH and high temperature. Reports of a few methanotrophs (Dunfield et al., 2007; Pol et al., 2007; Islam et al., 2008), which are capable of thriving in acidic (pH 0–4) and high temperature (∼65°C) environments, have led to the proposed family *Methylacidiphilaceae* (phylum: Verrucomicrobia). The global distribution and genetic relatedness of species within this family are still not well-understood. Given the distance, water bodies, and other geographic boundaries that exist between geothermal habitats worldwide, it is not known how conserved this taxon may be. From this study, all six previously uncharacterized isolates (i.e., Fdl, Rib, Fur, Yel, Ice, Phi) are shown to be closely related to one another as determined by 16S rRNA gene sequence comparisons as well as to the three previously described isolates of the family, specifically: SolV (Pol et al., 2007), V4 (Dunfield et al., 2007), and Kam1 (Islam et al., 2008). In addition to sharing high 16S rRNA gene sequence similarity (≥98.6%), morphological (i.e., size and shape) and physiological (e.g., growth rates and metabolism) properties of these newly reported geographically-distinct isolates suggest close relatedness.

However, DNA-DNA hybridization (DDH) studies indicate that not all of these new isolates are likely to be of the same species (and, perhaps, not even of the same genus). Indeed, employing the standard DDH cut-off (≥70%) for “same species” determination (Wayne et al., 1987; Goris et al., 2007), it appears isolates Fur, Fdl, Rib, SolV, and Ice are all geographic variants or *geovars* of the same species (see Table 2). Despite geographic barriers (i.e., waterways) between the Fur/Fdl/Rib group and SolV and Ice, all five geovars are within the same tectonic plate (i.e., the Eurasian Plate). Notably, isolate Ice originates from a geothermal region that borders the Eurasian Plate and the North American Plate (see Figure 1). By the proposed 16S rRNA gene identity threshold of >98.6% for “same species” determination (Chun et al., 2018), Yel may be the same species as Ice and the rest of the Eurasian group. However, the DDH value between isolate Yel and Ice is well below the standard 70% cut-off for DDH-based same species determination. Still, Yel shows a higher DDH value to Ice (61%) and other Eurasian Plate isolates (55–60.7%) than to isolates from other tectonic plate areas – V4 (20.4%), Phi (20.1%), and Kam1 (50.4%). Although the sampling site from which Phi was derived also sits at the edge of the Eurasian Plate (adjacent to the Philippine Sea Plate), DDH shows more distant relatedness between Phi and members of the Eurasian Plate group (i.e., Fur, Fdl, Rib, Ice, and SolV). Moreover, it appears that isolates Phi, V4, Kam1, and Yel, which each are derived from geothermal features in distinct tectonic plate regions are also more distantly-related in pairwise comparisons to one another than the within-group pairwise comparisons of isolates in Eurasian Plate clade. In short, this system exhibits biogeographic structure and tectonic boundaries may play a role in allopatric evolution and speciation. The proposed relationships between geography and genetic divergence are further supported by examination of phylogenetics of taxon-specific genes (i.e., *pmoAB2*) and concatenated contigs (i.e., 16S-ITS-23S) (see Figure 3–5).

Based on these results, we suggest that isolates Fur, Rib, and Fdl are the same species and that SolV and Ice are also geovars of that same species. However, Yel, Phi, V4, and Kam1 are all distinct species, perhaps within the same genus. We further suggest that species divergence from a common ancestor is the result of allopatric evolution within geographically-distinct geothermal habitats, which are not only separated by distance and water bodies but also by tectonic plate boundaries. As further isolates are collected from other regions in the world, we anticipate that novel species of the genus *Methylacidiphilium* will be characterized and that descriptions of additional isolates from other tectonic regions may resolve multiple genera within the family *Methylacidiphilaceae*.

## Author Contributions

H-AE, N-KB, and RC conceived and designed the experiments and did the sampling. H-AE and NS performed the experiments under supervision of N-KB. All authors interpreted the results and contributed in writing the manuscript.

## Conflict of Interest Statement

The authors declare that the research was conducted in the absence of any commercial or financial relationships that could be construed as a potential conflict of interest.

## Footnotes

1 https://www.google.com/maps/

2 http://www.gatc-biotech.com

3 http://rast.nmpdr.org/

4 http://www.ncbi.nlm.nih.gov/genome/annotation_prok/

5 http://ggdc.dsmz.de/

6 http://darlinglab.org/mauve/mauve.html

7 https://www.dsmz.de/home.html

8 http:/www.biogenes.de
