# Supplementary material for: Global Biogeographic Distribution Patterns of Thermoacidophilic Verrucomicrobia Methanotrophs Suggest Allopatric Evolution
Source: Front Microbiol. 2019 May 29;10:1129. doi: 10.3389/fmicb.2019.01129 (PMC6549252; doi:10.3389/fmicb.2019.01129)
Supplement: Supplementary file 1 [file Data_Sheet_1.PDF]

Global Biogeographic Distribution Patterns of Thermoacidophilic Verrucomicrobia Methanotrophs Suggest Allopatric Evolution

Helge-André Erikstad, Ruben Michael Ceballos, Natalie Bennett Smestad and

Nils-Kåre Birkeland

SUPPLEMENTARY FIGURES AND TABLES

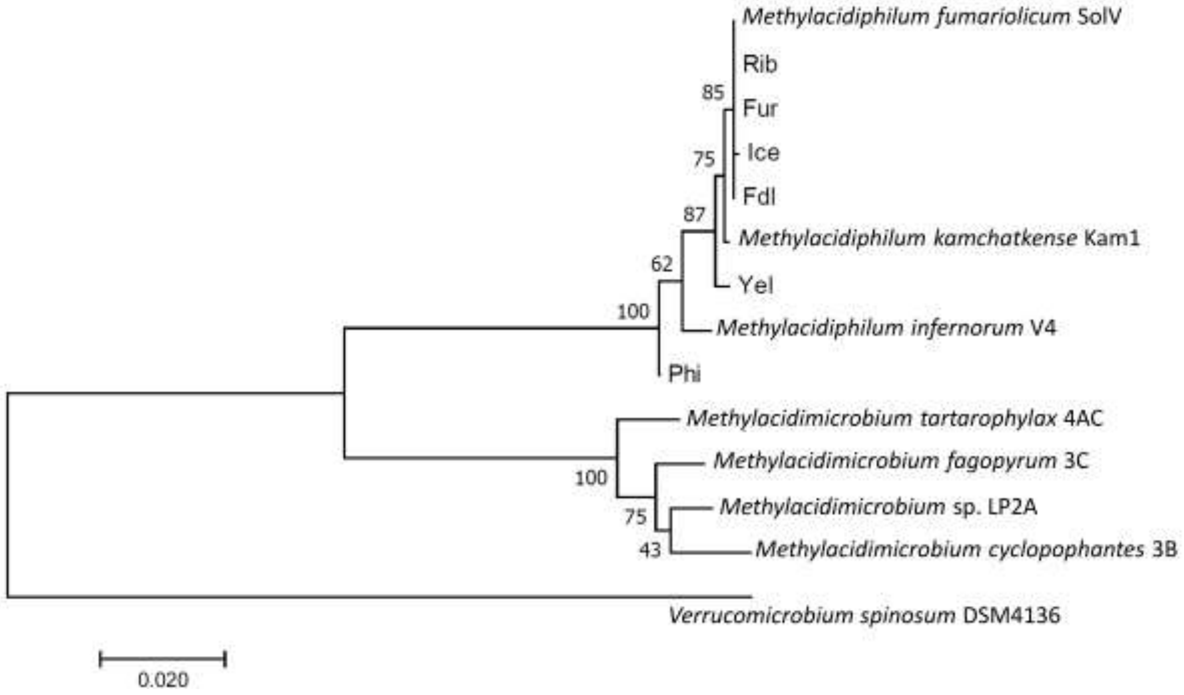

FIGURE S1 Phylogenetic analysis of *Methylacidiphilum* and *Methylacidimicrobium* spp. and novel strains by the Maximum Likelihood method using 16S rRNA genes. *Verrucomicrobium spinosum* was used as outgroup. The percentage of trees in which the associated taxa clustered together is shown next to the branches. The tree is drawn to scale, with branch lengths measured as the number of substitutions per site. All positions containing gaps and missing data were eliminated. Accession numbers for strains 4AC, 3C, LP2A, 3B and DSM4136 are NR_126314.1, NR_126313.2, JAFS00000000.1, NR_126315.2 and NR_026266.1, respectively.

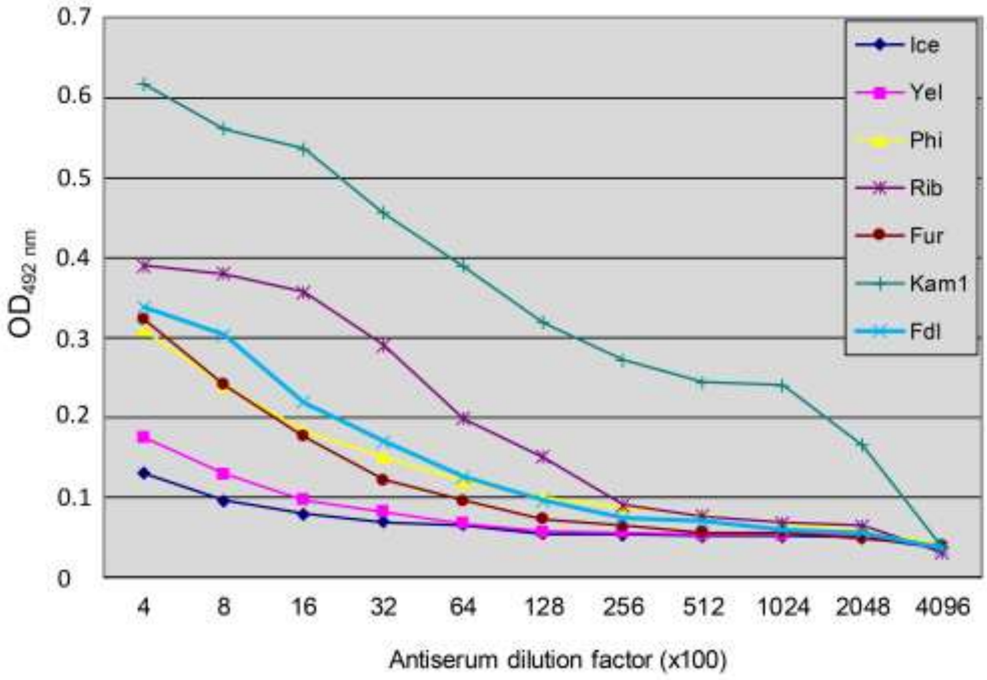

FIGURE S2 Cross reactions of 7 different *Methylacidiphilum* spp. strains (Ice, Yel, Phi, Rib, Fur, Kam1 and Fdl**)** towards rabbit antiserum raised against *M. kamchatkense* strain Kam1, as determined by ELISA and absorbance readings at 492nm. Color codes for antigens are given in the insert.

TABLE S1 Chemical composition of the sampled hot spring source waters*

Source	Major elements (ppm)									Minor elements (ppb)			
	Al	Ca	Fe	K	Mg	Mn	Na	S	Si	Ba	Cu	Zn	Total lanthanides
Krysuvik	5.2	34.2	8.1	1.4	13.4	0.3	17.4	74.6	36.9	12.0	ND	14.9	8.9
Makiling	37.6	18.5	101.9	4.6	38.6	1.2	3.9	409.8	169.0	3.4	ND	256.1	27.4
Furnas	14.0	27.5	14.4	45.0	13.1	2.0	64.0	238.2	178.0	19.5	16.9	253.7	ND
Ribeira G.†	43.0	24.5	20.4	26.0	7.9	1.0	38.0	410.1	134.4	18.5	15.8	626.4	ND
F. das Lagos‡	nd	6.2	0.05	23.0	11.6	0.001	26.0	14.8	0.04	57.0	9.1	150.3	ND
Kamchatka	nd	27.5	0.9	11.7	4.1	0.5	256.0	80.5	119.3	101.0	nd#	nd	ND

*nd, not detected; ND, not determined
#detection limit: 5 ppb
†Ribeira Grande
‡Furnas das Lagos

TABLE S2 Pairwise average nucleotide identity (ANI) values for the genome sequences of all thermoacidophilic methanotrophic Verrucomicrobia isolates*

Strain designation	SolV	Fur	Rib	Fdl	Ice	Yel	Phi	V4	Kam1
**SolV**	100								
**Fur**	99.97	100							
**Rib**	99.98	99.99	100						
**Fdl**	99.99	100	100	100					
**Ice**	99.99	100	100	99.99	100				
**Yel**	94.17	94.20	94.18	93.70	94.14	100			
**Phi**	75.65	75.57	75.55	75.58	75.67	74.72	100		
**V4**	75.20	75.31	75.07	74.35	74.31	74.27	77.69	100	
**Kam1**	92.93	92.91	92.93	92.30	92.92	92.5	75.31	73.76	100

*Determined using the ANI Calculator at http://enve-omics.ce.gatech.edu/ani/.

TABLE S3 Cellular fatty acid profiles of seven methanotrophic Verrucomicrobia strains given as percentage of the total fatty acid content[#]

Fatty acid	Ice	Yel	Phi	Fdl	Rib	Fur	Kam1*	V4*	SolV*
a13:1 12-13	0.1	0.1	0.1	-	0.1	0.1	-	-	-
13:0	-	-	-	-	-	0.1	-	0.2	-
i14:0	18.0	22.2	16.4	18.4	15.5	17.3	22.4	7.2	11.6
14:0	5.8	9.3	10.8	7.2	9.8	6.6	7.0	12.6	2.1
i15:0	3.0	2.5	3.1	3.3	2.7	2.5	2.8	1.7	2.1
a15:0	29.0	25.9	25.3	27.0	27.1	29.3	31.9	**12.9**	24.6
15:0	**5.7**	0.2	-	0.1	0.1	0.2	0.4	2.5	-
i16:0	**10.8**	4.0	5.6	4.6	4.0	4.5	3.9	1.4	3.2
16:0	0.3	7.6	11.1	12.5	14.1	12.5	9.9	14.4	10.5
a16:0	-	-	-	-	-	0.1	-	-	-
16:0 3OH	0.7	4.8	4.8	4.9	4.6	4.6	1.2	-	-
16:1 ω7c	-	-	-	-	-	-	-	0.1	-
i17:0	1.9	0.1	0.4	0.3	0.2	0.2	-	-	0.3
a17:0	0.5	0.9	1.9	1.4	1.5	1.6	1.2	0.4	1.1
17:0	**5.0**	0.7	0.2	0.8	0.4	0.8	1.5	**6.1**	0.5
17:0 3OH	-	0.1	-	-	-	0.1	-	-	-
i18:0	0.4	0.3	0.8	0.5	0.4	0.5	0.3	-	1.8
18:1 ω9c	0.2	0.4	0.1	0.3	0.1	0.4	1.7	0.7	-
18:0	18.4	20.5	19.3	18.8	19.0	18.6	13.7	**38.8**	**42.1**
18:0 10MeC	-	-	-	-	-	-	-	0.3	-
18:0 3OH	0.1	-	0.2	-	0.1	0.1	-	-	-
18:1 ω7c	-	-	-	-	0.1	-	-	0.2	-
19:0	-	0.1	-	-	-	-	-	-	-
20:0	-	0.3	0.1	-	-	-	-	-	-

*Data from Op den Camp et al. (2009)

[#] i, iso; a, anteiso; -, not detected; values in bold deviate significantly from other isolates.
